# Antibody-mediated immune responses and cardiovascular disease: a Mendelian randomization study

**DOI:** 10.3389/ebm.2026.10945

**Published:** 2026-05-12

**Authors:** Yi Li, Jiuqi Fan, Lirong Wu

**Affiliations:** Department of Cardiology, Guiqian International General Hospital, Guiyang, China

**Keywords:** antibody-mediated immune responses, cardiovascular diseases, infectious agents, Mendelian randomization, immune traits

## Abstract

Cardiovascular diseases represent the leading cause of global mortality and disability, posing a severe threat to human health. Accumulating evidence suggests that antigen–antibody–mediated immune responses may be involved in the pathogenesis of various cardiovascular conditions; however, whether these associations reflect causal relationships has long remained unclear. To address this question, we conducted a bidirectional two-sample Mendelian randomization study leveraging summary-level data from genome-wide association studies. In this analysis, 46 antibody-mediated immune traits were evaluated as exposures, and 11 cardiovascular outcomes, including aortic aneurysm, aortic valve stenosis, atrial fibrillation, coronary artery disease, dilated cardiomyopathy, atrioventricular block, heart failure with reduced ejection fraction, hypertrophic cardiomyopathy, infective endocarditis, myocarditis, and pericarditis, were examined as outcomes. Our results revealed several significant causal associations: genetically predicted higher levels of Epstein–Barr virus EBNA-1 antibodies were associated with increased risks of myocarditis and aortic valve stenosis, while elevated VCA p18 antibody levels were linked to a higher risk of myocarditis. Furthermore, increased antibody levels against BK polyomavirus VP1 were causally associated with greater risks of aortic valve stenosis and dilated cardiomyopathy. In contrast, higher levels of antibodies against varicella-zoster virus glycoproteins and human herpesvirus 6 IE1B were associated with reduced risks of myocarditis and aortic aneurysm, respectively. These findings not only help clarify the causal role of immune-mediated mechanisms in cardiovascular pathogenesis but also provide a theoretical foundation for the future development of immune-targeted strategies for prevention and treatment.

## Impact statement

Our findings deepen the understanding of immune-mediated mechanisms of cardiac injury and provide genetic evidence supporting precision-medicine interventions. However, the clinical feasibility of targeting antibody responses requires further validation in future studies.

## Introduction

Cardiovascular disease (CVD) is a principal driver of global mortality and disability. The Global Burden of Disease study indicates that CVD generally represents about 30%–35% of total deaths worldwide [1]. Therefore, identifying factors that influence the progression of CVD is of paramount importance. Current evidence implicates viral infections, dysregulated immune function, genetic predisposition, lifestyle factors and environmental exposures as important contributors to CVD risk [2–5]. Histopathological studies show increased infiltration of immune cells in CVD pathological tissue suggesting that immune-response–driven matrix injury is implicated throughout the course of CVD development [6–9]. A Mendelian randomization (MR) studies have shown that various antibody-mediated immune responses are risk factors for myocarditis [10]. Immune cells recognize antigens and produce specific antibodies that limit viral spread and clear pathogens via neutralization, opsonization, complement activation, and Fc receptor–mediated effector functions [11, 12]. As key effector molecules of the immune response, antibody levels can serve both as biomarkers of infectious exposure and as pathophysiological clues linking infection to noncommunicable diseases (for example, molecular mimicry, immune complex deposition, and dysregulated immune modulation) [13–15]. Nevertheless, the potential pathophysiological links between infectious agents and CVD remain unclear, and whether a true causal relationship exists is still unclear. Guillaume Butler-Laporte et al. identified genetic variants related to antibody-mediated immune responses through genome-wide association studies [16]. Unlike polygenic risk scores—which aggregate genetic susceptibility for risk prediction but cannot discern directionality or causality.MR is a genetic epidemiological approach specifically designed for causal inference, leveraging genetic variants as instrumental variables to estimate the causal effect of an exposure on an outcome while minimizing confounding and reverse causation bias [17, 18]. Thus, applying MR creates a new opportunity to investigate causal relationships between antibody-mediated immune responses and CVD. In this study, we performed bidirectional, two-sample Mendelian randomization analyses to systematically assess causal links between antibody-mediated immune responses and eight CVD, with the aim of advancing understanding of immune-mediated cardiovascular injury mechanisms and offering genetic evidence to support future precision immunotherapy strategies.

## Materials and methods

**FIGURE 1:**
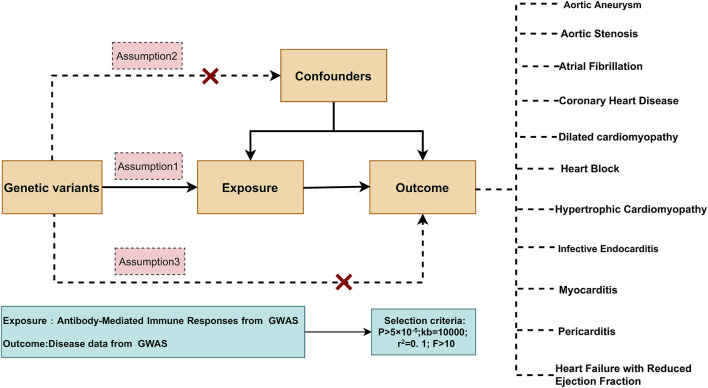
Design of the two-sample mendelian randomization analysis.

We first obtained summary-level data from published genome-wide association studies (GWAS), comprising 46 antibody-mediated immune response traits and eleven common cardiovascular diseases (aortic aneurysm, aortic valve stenosis, coronary artery disease, hypertrophic cardiomyopathy, dilated cardiomyopathy, infective endocarditis, myocarditis, pericarditis, atrial fibrillation, heart block, and reduced ejection fraction heart failure) (Table 1). We then applied two-sample MR to systematically evaluate the potential causal effects of antibody-mediated immune responses on these cardiovascular outcomes (Figure 1).

**TABLE 1:** GWAS traits used as exposures (antibody responses) and outcomes (cardiovascular diseases) in the MR analysis, with corresponding GWAS Catalog IDs (GCST).

Category	GWAS ID	Trait name
Exposure	GCST90006909	Human herpes virus 7 U14 antibody levels
Exposure	GCST90006910	Anti-helicobacter pylori IgG seropositivity
Exposure	GCST90006911	*Helicobacter pylori* CagA antibody levels
Exposure	GCST90006912	*Helicobacter pylori* Catalase antibody levels
Exposure	GCST90006913	*Helicobacter pylori* GroEL antibody levels
Exposure	GCST90006914	*Helicobacter pylori* OMP antibody levels
Exposure	GCST90006915	*Helicobacter pylori* UREA antibody levels
Exposure	GCST90006916	*Helicobacter pylori* VacA antibody levels
Exposure	GCST90006917	Anti-herpes simplex virus 1 IgG seropositivity
Exposure	GCST90006918	Herpes simplex virus 1 mgG-1 antibody levels
Exposure	GCST90006919	Anti-herpes simplex virus 2 IgG seropositivity
Exposure	GCST90006920	Herpes simplex virus 2 mgG-1 antibody levels
Exposure	GCST90006921	Anti-polyomavirus 2 IgG seropositivity
Exposure	GCST90006922	Polyomavirus 2 JC VP1 antibody levels
Exposure	GCST90006923	Anti-merkel cell polyomavirus IgG seropositivity
Exposure	GCST90006924	Merkel cell polyomavirus VP1 antibody levels
Exposure	GCST90006925	Anti-toxoplasma gondii IgG seropositivity
Exposure	GCST90006926	Toxoplasma gondii p22 antibody levels
Exposure	GCST90006927	Toxoplasma gondii sag1 antibody levels
Exposure	GCST90006928	Anti-varicella zoster virus IgG seropositivity
Exposure	GCST90006929	Varicella zoster virus glycoproteins E and I antibody levels
Exposure	GCST90006885	BK polyomavirus VP1 antibody levels
Exposure	GCST90006886	Anti-chlamydia trachomatis IgG seropositivity
Exposure	GCST90006887	*Chlamydia trachomatis* momp A antibody levels
Exposure	GCST90006888	*Chlamydia trachomatis* momp D antibody levels
Exposure	GCST90006889	*Chlamydia trachomatis* pGP3 antibody levels
Exposure	GCST90006890	*Chlamydia trachomatis* PorB antibody levels
Exposure	GCST90006891	*Chlamydia trachomatis* tarp-D F1 antibody levels
Exposure	GCST90006892	*Chlamydia trachomatis* tarp-D F2 antibody levels
Exposure	GCST90006893	Anti-cytomegalovirus IgG seropositivity
Exposure	GCST90006894	Cytomegalovirus pp28 antibody levels
Exposure	GCST90006895	Cytomegalovirus pp52 antibody levels
Exposure	GCST90006896	Cytomegalovirus pp150 antibody levels
Exposure	GCST90006897	Anti-Epstein-barr virus IgG seropositivity
Exposure	GCST90006898	Epstein-barr virus EA-D antibody levels
Exposure	GCST90006899	Epstein-barr virus EBNA-1 antibody levels
Exposure	GCST90006900	Epstein-barr virus VCA p18 antibody levels
Exposure	GCST90006901	Epstein-barr virus ZEBRA antibody levels
Exposure	GCST90006902	Anti-human herpes virus 6 IgG seropositivity
Exposure	GCST90006903	Anti-human herpes virus 6 E1A IgG seropositivity
Exposure	GCST90006904	Human herpes virus 6 IE1A antibody levels
Exposure	GCST90006905	Anti-human herpes virus 6 IE1B IgG seropositivity
Exposure	GCST90006906	Human herpes virus 6 IE1B antibody levels
Exposure	GCST90006907	Human herpes virus 6 p101k antibody levels
Exposure	GCST90006908	Anti-human herpes virus 7 IgG seropositivity
Exposure	GCST90006884	Anti-BK polyomavirus IgG seropositivity
Outcome	GCST90480203	Aortic aneurysm
Outcome	GCST90480118	Aortic stenosis
Outcome	GCST90624411	Atrial fibrillation
Outcome	GCST90480129	Coronary heart disease
Outcome	GCST90018834	Dilated cardiomyopathy
Outcome	GCST90480159	Heart block
Outcome	GCST90018861	Hypertrophic cardiomyopathy
Outcome	GCST90480146	Infective endocarditis
Outcome	GCST90436090	Myocarditis
Outcome	GCST90480145	Pericarditis
Outcome	GCST90480182	Reduced ejection fraction heart failure

### Data sources

The summary data of antibody mediated immune response comes from the antibody characteristic GWAS study by Butler Laporte et al [16]. This is the largest and only publicly available GWAS to date that systematically characterizes genetic determinants of antigen-specific antibody levels across a broad panel of infectious agents. The study analyzed 46 serological phenotypes—derived from mean fluorescence intensity measurements of serum antibodies against 13 pathogens—in approximately 8,735 White British participants from the UK Biobank. Genetic instruments for our Mendelian randomization analyses were drawn exclusively from this comprehensive resource^1^. Summary statistics for the cardiovascular disease outcomes were retrieved from the OpenGWAS platform^2^.

### Instrumental variable selection

The validity of causal inference in this Mendelian Randomization (MR) study is grounded on three fundamental assumptions regarding genetic variants: (1) the instrumental variables (IVs) are robustly associated with the exposure (antibody-mediated immune responses); (2) the IVs are independent of any confounding factors; (3) the IVs influence the outcome (various cardiac diseases) solely through the exposure pathway, without pleiotropic effects.

To identify appropriate IVs, single nucleotide polymorphisms (SNPs) associated with antibody-mediated immune traits were selected based on a genome-wide significance threshold of *P* < 5 × 10^−5^. Independent SNPs were retained after performing LD clumping with an r^2^ threshold of 0.1 within a 10,000 kb window.

To mitigate horizontal pleiotropy and reduce bias, the MR-PRESSO (Mendelian Randomization Pleiotropy RESidual Sum and Outlier) test was employed. SNPs identified as outliers (*P* < 0.05) were iteratively removed until the global pleiotropy test indicated no residual pleiotropic effect (*P* > 0.05). Furthermore, SNPs directly associated with the outcome at *P* < 0.05 were excluded to prevent confounding. Only SNPs with F-statistics greater than 10 were retained to avoid weak instrument bias.

### Statistical analysis

Causal effects were estimated using five complementary MR methods: inverse variance weighted (IVW) as the primary approach due to its statistical efficiency, supplemented by maximum likelihood, MR-Egger regression, weighted median, and weighted mode methods to ensure robustness. Reverse MR analyses were conducted using identical procedures to evaluate potential reverse causal relationships.

All analyses were performed in R software (version 4.3.1) utilizing the TwoSampleMR (version 0.5.7) and MR-PRESSO (version 1.0) packages.

## Results

### Relationship between antigen–antibody responses and aortic aneurysm

**FIGURE 2:**
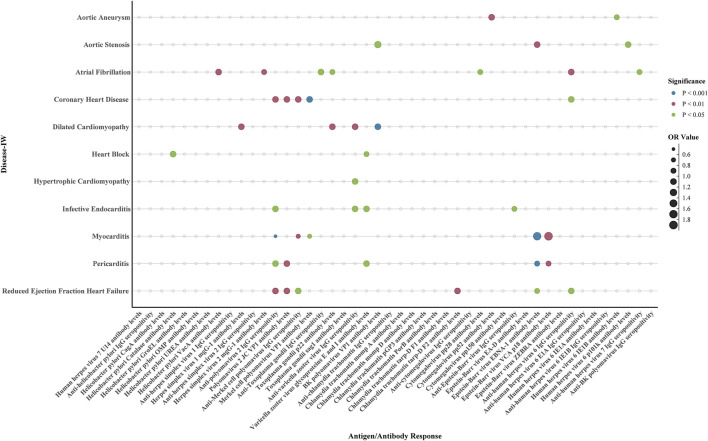
Causal estimates from Mendelian randomization analyis of antibody-mediated immune responses on cardiovascular diseases. Each dot represents the inverse-variance weighted (IVW) odds ratio (OR) estimate for the association between a specific antigen/antibody response and a cardiovascular disease. The size of the dot corresponds to the magnitude of the OR, with larger dots indicating stronger effects. Color indicates statistical significance: blue (P < 0.001), red (P < 0.01), and green (P < 0.05). Antibody responses associated with multiple cardiovascular outcomes are underlined in red. IVW: inverse-variance weighted; MR: Mendelian randomization.

**FIGURE 3:**
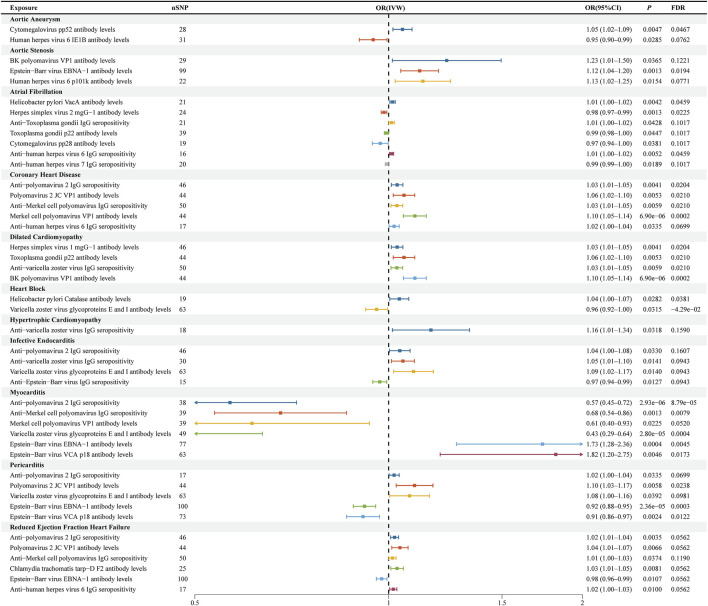
Forest plots for causal effects of antibody-mediated immune responses on cardiovascular diseases. The horizontal bars correspond to the estimated odds ratios (ORs) with 95% confidence intervals (CIs) using the inverse-variance weighted (IVW) method. MR: Mendelian randomization; OR: odds ratio; CI: confidence interval.

This study identified two antibody-mediated immune responses with causal associations to aortic aneurysm at the nominal significance level (P < 0.05). Both associations showed no evidence of significant heterogeneity or horizontal pleiotropy in sensitivity analyses (Supplementary Tables S2, S3). Specifically, genetically predicted higher levels of Cytomegalovirus pp52 antibody were associated with an increased risk of aortic aneurysm (OR = 1.05, 95% CI 1.02–1.09, P = 0.005). Conversely, higher levels of Human herpes virus 6 IE1B antibody were associated with a reduced risk of aortic aneurysm (OR = 0.95, 95% CI 0.90–0.99, P = 0.028) (Figures 2, 3; Supplementary Table S1). The Wald ratio estimates for individual SNPs are presented in Supplementary Table S4.

### Relationship between antigen–antibody responses and aortic valve stenosis

This study identified three antibody-mediated immune responses with causal associations with aortic valve stenosis at the nominal significance level (P < 0.05). All three showed no evidence of significant heterogeneity or horizontal pleiotropy in sensitivity analyses (Supplementary Tables S2, S4). Specifically, genetically predicted higher levels of BK polyomavirus VP1 antibody (OR = 1.23, 95% CI 1.01–1.50, P = 0.037), Epstein-Barr virus EBNA-1 antibody (OR = 1.12, 95% CI 1.04–1.20, P = 0.001), and Human herpes virus 6 p101k antibody (OR = 1.13, 95% CI 1.02–1.25, P = 0.015) were associated with an increased risk of aortic valve stenosis (Figures 2, 3; Supplementary Table S1). The Wald ratio estimates for individual SNPs are presented in Supplementary Table S4.

### Relationship between antigen–antibody responses and coronary artery disease

This study identified five antibody-mediated immune responses with causal associations to coronary heart disease at the nominal significance level (P < 0.05). Sensitivity analyses indicated that four of these associations showed no evidence of significant heterogeneity or horizontal pleiotropy (Supplementary Tables S2, S3). Specifically, genetically predicted higher levels of Merkel cell polyomavirus VP1 antibody (OR = 1.10, 95% CI 1.05–1.14, P < 0.001), Polyomavirus 2 JC VP1 antibody (OR = 1.06, 95% CI 1.02–1.10, P = 0.005), Anti-polyomavirus 2 IgG seropositivity (OR = 1.03, 95% CI 1.01–1.05, P = 0.004), and Anti-human herpes virus 6 IgG seropositivity (OR = 1.02, 95% CI 1.00–1.04, P = 0.034) were robustly associated with an increased risk of coronary heart disease (Figures 2, 3; Supplementary Table S1). However, the association with Anti-Merkel cell polyomavirus IgG seropositivity (IVW: OR = 1.03, 95% CI 1.01–1.05, P = 0.006) exhibited both significant heterogeneity (Q P = 0.022) and evidence of horizontal pleiotropy (MR-Egger intercept P = 0.017), suggesting that this estimate may be biased. The Wald ratio estimates for individual SNPs are presented in Supplementary Table S4.

### Relationship between antigen–antibody responses and dilated cardiomyopathy

This study identified four antibody-mediated immune responses with causal associations to dilated cardiomyopathy at the nominal significance level (P < 0.05). All associations showed no evidence of significant heterogeneity or horizontal pleiotropy in sensitivity analyses (Supplementary Tables S2, S3). Specifically, genetically predicted higher levels of BK polyomavirus VP1 antibody (OR = 1.10, 95% CI 1.05–1.14, P < 0.001), Toxoplasma gondii p22 antibody (OR = 1.06, 95% CI 1.02–1.10, P = 0.005), Herpes simplex virus 1 mgG-1 antibody (OR = 1.03, 95% CI 1.01–1.05, P = 0.004), and Anti-varicella zoster virus IgG seropositivity (OR = 1.03, 95% CI 1.01–1.05, P = 0.006) were all associated with an increased risk of dilated cardiomyopathy (Figures 2, 3; Supplementary Table S1). The Wald ratio estimates for individual SNPs are presented in Supplementary Table S4.

### Relationship between antigen–antibody responses and hypertrophic cardiomyopathy

This study identified one antibody-mediated immune response with a causal association to hypertrophic cardiomyopathy. The analysis showed no evidence of heterogeneity or horizontal pleiotropy (Supplementary Tables S2, S3). Specifically, genetically predicted Anti-varicella zoster virus IgG seropositivity was associated with an increased risk of hypertrophic cardiomyopathy (OR = 1.16, 95% CI 1.01–1.34, P = 0.032). The Wald ratio estimates for individual SNPs are presented in Supplementary Table S4.

### Relationship between antigen–antibody responses and infective endocarditis

This study identified four antibody-mediated immune responses with causal associations to infective endocarditis at the nominal significance level (P < 0.05). All associations showed no evidence of significant heterogeneity or horizontal pleiotropy in sensitivity analyses (Supplementary Tables S2,S3). Specifically, genetically predicted higher levels of Varicella zoster virus glycoproteins E and I antibody (OR = 1.09, 95% CI 1.02–1.17, P = 0.014), Anti-varicella zoster virus IgG seropositivity (OR = 1.05, 95% CI 1.01–1.10, P = 0.014), and Anti-polyomavirus 2 IgG seropositivity (OR = 1.04, 95% CI 1.00–1.08, P = 0.033) were associated with an increased risk of infective endocarditis. Conversely, Anti-Epstein-Barr virus IgG seropositivity was associated with a reduced risk (OR = 0.97, 95% CI 0.94–0.99, P = 0.013). The Wald ratio estimates for individual SNPs are presented in Supplementary Table S4.

### Relationship between antigen–antibody responses and myocarditis

This study identified six antibody-mediated immune responses with causal associations to myocarditis at the nominal significance level (P < 0.05). All associations showed no evidence of significant heterogeneity or horizontal pleiotropy in sensitivity analyses (Supplementary Tables S2,S3). Specifically, genetically predicted higher levels of Epstein–Barr virus EBNA-1 antibody (OR = 1.73, 95% CI 1.28–2.36, P < 0.001) and Epstein–Barr virus VCA p18 antibody (OR = 1.82, 95% CI 1.20–2.75, P = 0.005) were associated with an increased risk of myocarditis. Conversely, Anti-polyomavirus 2 IgG seropositivity (OR = 0.57, 95% CI 0.45–0.72, P < 0.001), Anti-Merkel cell polyomavirus IgG seropositivity (OR = 0.68, 95% CI 0.54–0.86, P = 0.001), Merkel cell polyomavirus VP1 antibody levels (OR = 0.61, 95% CI 0.40–0.93, P = 0.023), and Varicella zoster virus glycoproteins E and I antibody levels (OR = 0.43, 95% CI 0.29–0.64, P < 0.001) were associated with a reduced risk of myocarditis (Figures 2, 3; Supplementary Table S1). The Wald ratio estimates for individual SNPs are presented in Supplementary Table S4.

### Relationship between antigen–antibody responses and pericarditis

This study identified five antibody-mediated immune responses with causal associations to pericarditis at the nominal significance level (P < 0.05). Sensitivity analyses indicated that four of these associations showed no evidence of significant heterogeneity or horizontal pleiotropy (Supplementary Tables S2, S3). Specifically, genetically predicted higher levels of Polyomavirus 2 JC VP1 antibody (OR = 1.10, 95% CI 1.03–1.17, P = 0.006), Anti-polyomavirus 2 IgG seropositivity (OR = 1.02, 95% CI 1.00–1.04, P = 0.034), Varicella zoster virus glycoproteins E and I antibody levels (OR = 1.08, 95% CI 1.00–1.16, P = 0.039), and Epstein–Barr virus VCA p18 antibody levels (OR = 0.91, 95% CI 0.86–0.97, P = 0.002) were robustly associated with pericarditis risk (Figures 2, 3; Supplementary Table S1).

However, the inverse association between Epstein–Barr virus EBNA-1 antibody levels and pericarditis (OR = 0.92, 95% CI 0.88–0.95, P < 0.001) exhibited both significant heterogeneity (Q P = 0.011) and evidence of horizontal pleiotropy (MR-Egger intercept P = 0.012), suggesting that this estimate may be subject to bias. The Wald ratio estimates for individual SNPs are presented in Supplementary Table S4.

### Relationship between antigen–antibody responses and heart failure with reduced ejection fraction

This study identified six antibody-mediated immune responses with causal associations to heart failure with reduced ejection fraction at the nominal significance level (P < 0.05). All associations showed no evidence of significant heterogeneity or horizontal pleiotropy in sensitivity analyses (Supplementary Tables S2, S4). Specifically, genetically predicted higher levels of Polyomavirus 2 JC VP1 antibody (OR = 1.04, 95% CI 1.01–1.07, P = 0.0066), Anti-polyomavirus 2 IgG seropositivity (OR = 1.02, 95% CI 1.01–1.04, P = 0.0035), Chlamydia trachomatis tarp-D F2 antibody (OR = 1.03, 95% CI 1.01–1.05, P = 0.0081), Anti-human herpes virus 6 IgG seropositivity (OR = 1.02, 95% CI 1.00–1.03, P = 0.0100), and Anti-Merkel cell polyomavirus IgG seropositivity (OR = 1.01, 95% CI 1.00–1.03, P = 0.0374) were associated with an increased risk of heart failure with reduced ejection fraction. Conversely, higher levels of Epstein–Barr virus EBNA-1 antibody (OR = 0.98, 95% CI 0.96–0.99, P = 0.0107) were associated with a reduced risk (Figures 2, 3; Supplementary Table S1). The Wald ratio estimates for individual SNPs are presented in Supplementary Table S4.

### Relationship between antigen–antibody responses and atrial fibrillation

This study identified seven antibody-mediated immune responses with causal associations to atrial fibrillation at the nominal significance level (P < 0.05). Sensitivity analyses indicated that four of these associations showed no evidence of significant heterogeneity or horizontal pleiotropy (Supplementary Tables S2, S3). Specifically, genetically predicted higher levels of *Helicobacter pylori* VacA antibody (OR = 1.01, 95% CI 1.00–1.02, P = 0.004), Anti-human herpes virus 6 IgG seropositivity (OR = 1.01, 95% CI 1.00–1.02, P = 0.005), Anti-Toxoplasma gondii IgG seropositivity (OR = 1.01, 95% CI 1.00–1.02, P = 0.043), and lower Anti-human herpes virus 7 IgG seropositivity (OR = 0.99, 95% CI 0.99–1.00, P = 0.019) were robustly associated with atrial fibrillation risk (Figures 2, 3; Supplementary Table S1). However, three inverse associations exhibited evidence of both heterogeneity and horizontal pleiotropy: Herpes simplex virus 2 mgG-1 antibody levels (OR = 0.98, 95% CI 0.97–0.99, P = 0.001; Q P = 0.046, Egger P = 0.035), Cytomegalovirus pp28 antibody levels (OR = 0.97, 95% CI 0.94–1.00, P = 0.038; Q P = 0.0002, Egger P = 0.0007), and Toxoplasma gondii p22 antibody levels (OR = 0.99, 95% CI 0.98–1.00, P = 0.045; Q P = 0.017, Egger P = 0.023). These latter estimates may be subject to bias and should be interpreted with caution. The Wald ratio estimates for individual SNPs are presented in Supplementary Table S4.

### Relationship between antigen–antibody responses and heart block

This study identified two antibody-mediated immune responses with causal associations to heart block at the nominal significance level (P < 0.05). Both associations showed no evidence of significant heterogeneity or horizontal pleiotropy in sensitivity analyses (Supplementary Tables S2, S3). Specifically, genetically predicted higher levels of *Helicobacter pylori* Catalase antibody were associated with an increased risk of heart block (OR = 1.04, 95% CI 1.00–1.07, P = 0.028). Conversely, higher levels of Varicella zoster virus glycoproteins E and I antibody were associated with a reduced risk of heart block (OR = 0.96, 95% CI 0.92–1.00, P = 0.031) (Figures 2, 3; Supplementary Table S1). The Wald ratio estimates for individual SNPs are presented in Supplementary Table S4.

### Relationship between cardiovascular diseases and antigen–antibody responses

**FIGURE 4:**
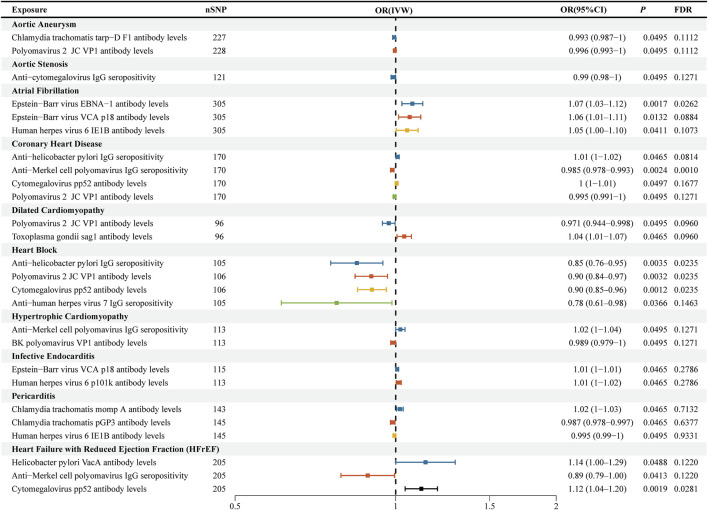
Forest plots for reverse Mendelian randomization analysis of cardiovascular diseases on antibody-mediated immune responses. The horizontal bars represent the inverse-variance weighted (IVW) odds ratios (ORs) with 95% confidence intervals (CIs) for the causal effect of each cardiovascular disease on specific antigen/antibody responses. Results are grouped by disease, and significant associations at FDR <0.05 are indicated in bold. IVW: inverse-variance weighted; OR: odds ratio; CI: confidence interval; FDR: false discovery rate.

Reverse Mendelian randomization analyses revealed several cardiovascular diseases with causal effects on antibody-mediated immune responses at the nominal significance level (P < 0.05). Most of these associations showed no evidence of significant heterogeneity or horizontal pleiotropy in sensitivity analyses (Supplementary Tables S6, S7). Specifically, genetically predicted atrial fibrillation was associated with elevated antibody levels against Epstein–Barr virus EBNA-1 (OR = 1.07, 95% CI: 1.03–1.12; P = 0.0017) and VCA p18 (OR = 1.06, 95% CI: 1.01–1.11; P = 0.013). Atrioventricular block was causally linked to reduced seropositivity to *Helicobacter pylori* (OR = 0.85, 95% CI: 0.76–0.95; P = 0.0035) and lower antibody levels against cytomegalovirus pp52 (OR = 0.90, 95% CI: 0.84–0.97; P = 0.0012) and polyomavirus 2 JC VP1 (OR = 0.90, 95% CI: 0.84–0.97; P = 0.0032). Additionally, HFrEF was associated with increased cytomegalovirus pp52 antibody levels (OR = 1.12, 95% CI: 1.04–1.20; P = 0.0019) and decreased anti–Merkel cell polyomavirus IgG seropositivity (OR = 0.89, 95% CI: 0.79–1.00; P = 0.041). These findings collectively suggest a modest but bidirectional interplay between cardiac pathology and humoral immune responses (Figure 4; Supplementary Table S5). The Wald ratio estimates for individual SNPs are presented in Supplementary Table S8.

## Discussion

The immune response to viral infection depends on key components such as the complement system, the coagulation cascade, and natural antibodies; these elements interact to form a complex network and are tightly regulated to maintain homeostasis and prevent immune dysregulation [19]. Antibody-mediated immune responses refer to antigen-specific antibodies produced by B cells that recognize and bind antigens and eliminate pathogens or toxins via neutralization, opsonization, complement activation, and Fc receptor–mediated cellular effector functions [11, 20, 21]. Antigen-specific immunity that limits viral spread and clears viruses is beneficial to the host; however, in some cases it can cause tissue damage [22]. The mechanisms by which viral infections cause myocardial injury remain debated, with three main hypotheses: immune cell–mediated myocardial destruction, autoimmune myocardial injury driven by circulating autoantibodies, and direct virus-induced cardiomyocyte cytotoxicity [23]. The prevailing view is that myocardial injury is mainly caused by virus-associated immune responses rather than direct viral action on cardiomyocytes [24].

Herpesviruses are widespread in humans, with cytomegalovirus (CMV),Epstein-Barr virus (EBV) and herpes simplex virus (HSV) being particularly common and implicated in various cardiovascular conditions. EBV infection is prevalent worldwide and can persist in the host, often lifelong. Acute EBV infection commonly causes acute cardiovascular injury manifested by myocarditis, pericardial involvement, and heart failure, whereas chronic EBV infection is more often associated with vascular lesions and chronic myocarditis [25, 26]. The mechanisms of EBV-induced cardiovascular injury can be categorized as direct and indirect. Screening of myocardial tissue from adults with idiopathic left ventricular dysfunction detected EBV in approximately 2% of cases [27], but quantitative analyses have not found an association between persistent EBV infection and idiopathic myocarditis [28]. In EBV-associated myocarditis, marked inflammatory infiltrates are observed despite lack of evidence for direct viral invasion of cardiomyocytes, suggesting indirect immune-mediated injury rather than direct cytotoxicity [29], Both EBV and HCMV lack specific receptors to infect cardiomyocytes directly and are frequently detected within inflammatory cells in the heart, further supporting an immune-mediated mechanism of cardiac damage [30]. This indirect, inflammation-driven pattern is also evident more broadly in the cardiovascular system. For example, in individuals carrying the T allele of VEGF rs3025039—which is associated with reduced VEGF expression—high titers of EBV early antigen IgG (EA-IgG) are significantly associated with an increased risk of atherosclerosis (OR = 1.88) [31]. Furthermore, cross-sectional studies have shown that higher burdens of antiviral IgG antibodies, such as EBV EA-IgG, are significantly linked not only to greater atherosclerosis risk but also to elevated levels of systemic inflammatory markers [32]. This suggests that cumulative pathogen-specific antibody responses may promote vascular injury through sustained low-grade systemic inflammation. In patients with acute myocardial infarction (AMI) who are seropositive for anti-EBV antibodies, EBV can trigger acute coronary events via the pro-inflammatory effects of its early protein dUTPase—even in the absence of active viral replication [33]. Similarly, among people with HIV, higher levels of CMV-specific IgG antibodies are significantly associated with atherosclerosis, obstructive coronary artery disease, and increased coronary plaque volume [34]. Another study showed that CMV seropositivity *per se* was associated with a modestly increased risk of secondary cardiovascular events in patients with coronary heart disease, but the risk was significantly amplified in those with diabetes (HR = 2.58), suggesting that CMV antibody positivity serves as an important inflammation-related risk marker in specific high-risk populations, such as individuals with diabetes [35]. This association likely stems from chronic immune activation driven by persistent CMV infection. In younger individuals, higher anti-CMV IgG titers are already linked to a pro-inflammatory state and early remodeling of memory T-cell subsets. In older adults, they primarily drive the expansion of terminally differentiated CD8^+^ effector memory cells and NK cells, reinforcing low-grade inflammation and adaptive immune skewing [36]. A study at the University of Minnesota reported a markedly higher incidence of CADat 2 years post–heart transplant among CMV-positive immunosuppressed patients (32%) compared with CMV-negative patients (10%) [37]. Serum antibody levels against varicella-zoster virus (VZV) are significantly associated with coronary atherosclerosis, and this association is modulated by host genetic background, specifically HLA-A alleles [38, 39]. In coxsackievirus B (CVB) infection, mice injected with CVB plus non-neutralizing antibodies exhibited viral titers in tissues that were one to two orders of magnitude higher than those in mice injected with virus alone. Concomitantly, these mice also showed more severe histopathological damage, particularly in the heart, suggesting that pre-existing antiviral antibodies may exacerbate cardiovascular disease through cross-reactive immune mechanisms [40]. Collectively, these findings indicate that virus-related antibody levels serve as markers of chronic immune activation and are positively correlated with cardiovascular disease burden, further supporting their potential role in the pathogenesis of atherosclerosis and coronary events. Our Mendelian randomization analysis demonstrates that genetically predicted higher levels of specific antiviral antibodies—rather than viral exposure per se—are differentially associated with increased cardiovascular disease risk. Elevated Epstein-Barr virus EBNA-1–specific IgG levels, a marker of long-term control of latent infection, were causally linked to aortic stenosis and myocarditis. Among beta herpesviruses, increased cytomegalovirus pp52-specific IgG levels were associated with aortic aneurysm, while higher human herpesvirus 6 p101k-specific IgG levels and anti-HHV-6 IgG seropositivity correlated with aortic stenosis and coronary heart disease, respectively. Additionally, greater herpes simplex virus type 1 glycoprotein G-1 (mgG-1)–specific IgG levels were positively associated with dilated cardiomyopathy. These findings further confirm that specific herpesvirus antibody levels are linked to cardiovascular disease risk, supporting their role in disease development and potential use in prevention strategies.

Serological positivity and pathogen detection rates for *Chlamydia* species are often higher in patients with coronary artery disease (CAD) than in control populations, suggesting a possible association with CAD [41]. In one comparative study that included 159 individuals with coronary artery disease, 71 with valvular heart disease, and 300 controls, seropositivity for *Chlamydia* exceeded 80% in the cohort [42]. *Chlamydia* pneumoniae IgG antibody levels are associated with the progression of aortic valve stenosis (AVS), suggesting that these antibodies may promote AVS development through sustained immune activation [43]. This notion is supported by multiple epidemiological studies showing that C. pneumoniae seropositivity is significantly linked to an increased risk of future cardiovascular events, particularly stroke [44, 45]. Moreover, C. pneumoniae IgG antibodies have been identified as an independent risk factor for coronary heart disease [46]. Mechanistically, persistent exposure to chlamydial antigens is thought to induce endothelial dysfunction and chronic vascular inflammation. Further evidence indicates that, in patients with coronary heart disease, seropositivity for *Chlamydia* LPS-specific IgA is strongly associated with elevated levels of inflammatory and endothelial activation markers, including IFNγ, IL-10, TNFα, sVCAM-1, and sE-selectin [47]. Consistent with this, Nazmi et al. reported that high antibody levels against C. pneumoniae are significantly correlated with systemic pro-inflammatory markers such as IL-6 and CRP, implicating chronic immune activation as a key pathway linking chlamydial immune responses to cardiovascular risk [48]. Beyond inflammation-driven pathways, autoimmune mechanisms may also contribute: for instance, the monoclonal antibody MAb 7B5, raised against chlamydial gG1, cross-reacts with human apolipoprotein B (ApoB), suggesting that molecular mimicry could induce autoantibodies against ApoB/LDL and thereby promote autoimmune responses in atherosclerosis [49]. Molecular mimicry may also contribute: actin and members of the HSP family share high homology with *Chlamydia* antigens (80.6%), suggesting cross-reactivity as a potential mechanism of tissue injury [50]. However, simple epitope similarity alone is insufficient to cause tissue damage; outcomes depend on HLA background, antigen presentation and MHC restriction, immune tolerance, and specific pathogen–host interactions [51]. Our Mendelian randomization analysis also identified a positive association within the *Chlamydia* family: genetically predicted higher levels of *Chlamydia trachomatis* Tarp-D F2–specific IgG antibodies were associated with an increased risk of heart failure with reduced ejection fraction. This finding highlights the need for further investigation into the role of chlamydial antigen-specific immune responses in cardiac outcomes.

In the Kitava population of Papua New Guinea, the high prevalence of *Treponema* infection is closely associated with elevated levels of naturally occurring cardioprotective autoantibodies—specifically IgM antibodies against phosphorylcholine (anti-PC IgM)—which have been linked to the low incidence of cardiovascular disease in this group, suggesting that certain infection-induced antibody responses may confer cardiovascular protection through immunomodulatory mechanisms [52]. This dual role of antiviral immunity is further illustrated in coxsackievirus B3 (CVB3)-induced myocarditis: high-titer neutralizing antibodies effectively block viral entry, suppress replication, and prevent myocardial injury, thereby exerting cardioprotective effects; in contrast, low-titer (sub-neutralizing) antibodies enhance viral uptake into macrophages via Fcγ receptors, promoting viral replication and exacerbating inflammation and tissue damage [53]. Supporting the importance of immune polarization, murine studies show that male mice develop severe myocarditis driven by Vγ4^+^ γδ T cell–mediated Th1/IFN-γ inflammatory responses, whereas female mice are protected through a Vγ1^+^ γδ T cell–dependent Th2/IL-4 pathway that promotes neutralizing antibody production [54, 55]. Seropositivity to common pathogens in adulthood—including enteroviruses, HSV, and *Chlamydia* pneumoniae—is associated with an increased risk of coronary heart disease (OR = 1.57–1.86), whereas a higher number of childhood infectious diseases (e.g., measles, chickenpox) correlates with reduced risk (OR = 0.86 per additional infection), highlighting the dual role of infections as both pro-inflammatory triggers and inducers of protective immune training [56]. Moreover, human cytomegalovirus (HCMV) can modulate humoral immunity by engaging IgG^+^ memory B cells via its glycoprotein gp34, suppressing their proliferation, plasma cell differentiation, and antibody secretion, thereby inducing a state of global B-cell hyperresponsiveness; however, altered gp34 phenotypes can instead promote more effective immune cell activation and restrict viral spread in HCMV-infected fibroblasts [33, 55]. In dilated cardiomyopathy, antibodies against cardiotropic viruses such as coxsackievirus B can cross-react with anti-myolemmal antibodies (AMLA); this molecular mimicry may confer protection by blocking pathogenic autoantibodies from binding to cardiomyocytes or by modulating complement activation, thereby attenuating myocardial injury [57]. The observed associations between certain antiviral antibodies and reduced cardiovascular disease (CVD) risk may not reflect a direct effect of the antibodies on the cardiovascular system. Instead, they likely signify that the host has successfully established a protective immune profile characterized by effective humoral immunity and anti-inflammatory responses. Such an immune profile may minimize virus-induced inflammatory cardiovascular damage while simultaneously modulating immune regulation or enabling cross-protective mechanisms that lower CVD risk. This provides an important immunological perspective for understanding the complex relationship between viral serological markers and CVD. Our Mendelian randomization analysis identified several protective associations between pathogen-specific antibody responses and cardiovascular outcomes. Genetically predicted higher levels of Epstein-Barr virus EBNA-1–specific IgG antibodies were significantly associated with a reduced risk of pericarditis (OR = 0.92, 95% CI: 0.88–0.95; p = 2.36 × 10^-5^) and heart failure with reduced ejection fraction (OR = 0.98, 95% CI: 0.96–0.99; p = 0.011). Similarly, elevated EBV VCA p18–specific IgG was linked to lower pericarditis risk (OR = 0.91, 95% CI: 0.86–0.97; p = 0.0024).Notably, seropositivity or higher antibody levels against multiple polyomaviruses conferred strong protection against myocarditis.

The characterization of pathogen-specific antigen–antibody interactions in cardiovascular disease opens promising avenues for translational applications. Beyond their role as diagnostic or prognostic biomarkers, these immune responses may inform the development of precision immunotherapies, for example, by harnessing protective antibody profiles to design monoclonal antibodies that mimic natural cardioprotective immunity or block pathogenic cross-reactivity. Furthermore, conserved microbial antigens capable of eliciting beneficial humoral responses could serve as candidates for therapeutic vaccines aimed at reprogramming maladaptive inflammation toward a more regulated, anti-inflammatory state. In drug discovery, molecular epitopes involved in protective versus pathogenic antibody binding may be leveraged to develop peptide-based inhibitors, small molecules, or tolerogenic platforms that selectively dampen autoimmune reactivity while preserving host defense. Ultimately, deciphering the fine specificity and functional consequences of antigen–antibody interactions could enable a new class of immune-guided interventions in cardiovascular medicine.

This study has several limitations. First, all samples were derived from individuals of European ancestry, and therefore the generalizability of our findings requires validation in cohorts from other ethnicities and geographic regions. Second, despite the corrections applied, the possibility of false-positive findings cannot be fully excluded. Third, our work provides epidemiological and genetic evidence of associations and potential causality but lacks sufficient cellular or animal model experiments to elucidate underlying molecular mechanisms, specific immune cell types involved, and detailed pathological processes.

## Conclusion

This study comprehensively evaluated causal relationships between antibody-mediated immune response phenotypes and eleven cardiovascular diseases, and confirmed these relationships were not confounded by other factors. We identified multiple antibody-mediated immune phenotypes that may influence aortic aneurysm, aortic valve stenosis, coronary artery disease, hypertrophic cardiomyopathy, dilated cardiomyopathy, infective endocarditis, myocarditis, pericarditis, atrial fibrillation, heart block, and reduced ejection fraction heart failure. Notably, antibody responses to Epstein–Barr virus (particularly EBNA-1 and VCA p18), cytomegalovirus pp52, and varicella zoster virus were robustly associated with altered risks of several cardiovascular conditions. These findings provide new evidence for the role of infections in cardiovascular disease, suggest novel approaches for screening and identifying high-risk individuals, and highlight the potential of antiviral immunity and inflammation-modulating strategies as targets for prevention and intervention.

## Data Availability

The data presented in this study are publicly available in the GWAS Catalog.

## Footnotes

1
https://www.ebi.ac.uk/gwas/studies/GCST90006909-GCST90006884

2
https://gwas.mrcieu.ac.uk/
